# Ninth International Medical Congress

**Published:** 1887-10

**Authors:** Nathan Smith Davis

**Affiliations:** President of the Congress


### THE DENTAL REGISTER.

VOL. XLI.] OCTOBER, 1887. [No. 10.

### COMMUNICATIONS.

### Ninth International Medical Congress.

Inaugural Address Delivered Monday, September 5, 1887, in Albaugh's Opera House, Washington, D. C.

BY NATHAN SMITH DAVIS, M.D., L.L.D.,

PRESIDENT OF THE CONGRESS.

*Gentlemen:*—It is my sad duty first to remind you that *death* has removed from among us *one* to whom, more than any other, we are indebted for the privilege of having the Ninth International Medical Congress in America. *One* whose urbanity, erudition, valuable contributions to medical literature and eminence as a teacher, caused him, not only to be universally regarded the most influential leader in all the preparatory work, but also *the one* unanimously designated to preside over your deliberations on this occasion.

That *one* was the late Professor Austin Flint, of New York, who was taken suddenly from his earthly labors early in 1886, before the work of preparation for this Congress had been half completed. The true nobility of his private and professional character, his eminent ability as a teacher, and above all the number and value of his contributions to the literature and art of medicine, had caused him to be known and esteemed by the profession in all countries. And as you all remember, while the shock of his death was fresh upon us, our loss seemed well-nigh irreparable; but though he has taken his departure, ripe in years and full of honors, yet the influence of his excellent example and his contributions to medical science remain, and will continue to exert their beneficent influence through all the generations to come.

With a full consciousness of my own deficiencies and still with a heart overflowing with gratitude, I thank you for the honor you have bestowed in selecting me to preside over the deliberations of this great and learned assembly. It is an honor that I appreciate as second to no other of a temporal nature because it has been bestowed, neither by conquest nor hereditary influence, nor yet by partisan strife, but by the free expression of your own choice.

Addressing myself now more directly to those here assembled, who have left homes and loved ones in other lands and encountered the fatigue and danger of traveling by sea and by land, in the name of the Medical Profession of this country I welcome you, not only to this beautiful city and the hospitality of its citizens as has been so admirably done already by the honorable representative of the Government, who has just taken his seat, but I cordially welcome you to the open arms and warm hearts of the medical men of this *whole country*, in whose name you were invited here three years since, and whose representatives are now here, side by side with you, gathered from the East, the West, the North, the South, as well as from the rugged mountains and fertile valleys of the Centre, to make good the promise implied by that invitation.

If they do not cause you to feel at home and happy, not only in the social circles and halls, devoted to the advancement of science, literature, and art in this city of our Nation's pride, but wherever you may choose to roam, from the rocky coast of New England on the Atlantic to the Golden Gate of the Pacific, it will be from no want of earnest disposition to do so.

And now, I not only thus welcome you from other lands, but I take great pleasure in greeting you one and all as leading representatives of a profession whose paramount object is the lessening of human suffering, by preventing, alleviating, or curing diseases wherever found, and in whatever class or grade of the human family. Nay, more, with profound reverence I greet you as a noble brotherhood, who in the practical pursuit of that one grand object, recognize no distinction of country, race, or creed, but bind up the wounds and assuage the pains of the rich and poor, ruler and ruled, Christian and pagan, friend and foe alike.

Not that every medical man does not love and defend his own country and fireside with as fervid a patriotism as the members of any other class of men; but as disease and pain are limited to no class or country, so is the application of his beneficent art limited only by the number of those suffering within his reach.

With a common object so beneficent in its nature, and opportunities for its practical pursuit so universal, it is but natural that you should be found searching for the most effectual means for the accomplishment of the one object of lessening human suffering, in every field of nature and in every department of human knowledge.

The living human body—the chief object of your solicitude—not only combines in itself the greatest number of elementary substances and the most numerous organs and varied functions, so attuned to harmonious action as to illustrate the operation of every law of physics, every known force in nature, and every step in the development of living matter from the simple aggregation of protoplasm constituting the germinal cell to the full-grown man, but it is placed in appreciable and important relations with the material objects and immaterial forces existing in the world in which he lives.

Hence a complete study of the living man, in health and disease, involves a thorough study, not only of his structure and functions but more or less of every element and force entering into the earth, the air, and the water with which he stands in constant relation.

The Medical Science of to-day, therefore, embraces not only a knowledge of the living man, but also of such facts, principles, and materials gathered from every other department of human knowledge as may increase your resources for preventing or alleviating his suffering and of prolonging his life.

The time has been, when medical studies embraced little else than the fanciful theories and arbitrary dogmas of a few leading minds, each of which became for the time the founder of a sect or so-called school of medicine, with his disciples more or less numerous. But with the development of general and analytical chemistry, of the several departments of Natural Science, of a more practical knowledge of physics, and the adoption of the inductive processes of reasoning, the age of theoretical dogmas and of medical sects blindly following some more plausible leader. passed away leaving but an *infinitesimal* shadow yet visible on the medical horizon.

So true is this, that in casting our mental vision, to-day, over the broad domain of medicine we see its votaries engaged, some searching for new facts and new materials; some studying new applications and better uses of facts and materials already known; some of them are in the dead house with scalpel and microscope, not only studying the position and relations of every part from the obvious bones and muscles to the smallest leucocyte, in health; but also every deviation caused by morbid action or disease. Some are searching the fields, the forest, the earth, and the air, both for more knowledge concerning the causes of disease and for additional remedial agents; some are in laboratories with crucible, test glass, and microscope analyzing every morbid product and every remedial agent, separating the active principles from the crude materials and demonstrating their action on living animals, while far the greater number are at the bed-side of the sick and wounded applying the knowledge gained by all other workers to the relief of human suffering. A more active, earnest, ceaseless, and beneficent field of labor is not open to your vision in any other direction or occupied by any other profession or class of men. And thus has the Science of Medicine become a vast aggregation of observed facts, many of them so related to each other as to permit practical deductions of permanent value, while many others remain isolated through incompleteness of investigations, and therefore liable to prompt, hasty, or even erroneous conclusions.

Indeed the most defective and embarrassing feature in the Science and Art of Medicine, at this time, is the rapid accumulation of facts furnished by the vast number of individual workers, each pushing investigations in some special direction without concert with his fellows, and without any adequate conception of the coincident lines of observation necessary to enable him to see the true bearing of the facts he evolves. Hence he is constantly mistaking mere coincidences for the relation of cause and effect, and the pages of our medical literature are being filled with hastily formed conclusions and rules of practice from inadequate data.

This results in part, at least, from the extent and variety of the fields of inquiry and the complexity of the problems presented for solution. For nowhere else within the realms of human thought, does the mind encounter problems requiring for their correct solution the consideration of a greater number of data, than in the study of etiology and pathology. To determine the appreciable conditions of the earth, air, and water of any country before, during, and after, the invasion of an epidemic disease long enough to include several consecutive visits of the same, is not possible for a single individual, nor for any number of observers acting separately or without concert.

Yet just this complete knowledge is necessary to enable us to separate the conditions that are merely coincident or accidental from those that are such constant accompaniments of the disease as to prove a necessary relation between them. And it is only by such persistent, coincident systematic observations by many individuals, each having a definite part, and the results carefully compared analytically and synthetically at proper intervals, that the real conditions and laws controlling the prevalence and severity of epidemics and endemics can be clearly demonstrated. It is not enough to discover the primary infection, or the *contagium vivum*, whether it be the bacillus of cholera, yellow fever, or tuberculosis, for abundant experience has shown that not one of these will extend its ravages in any community or country unless it finds there is a soil or pabulum congenial for its support and propagation.

It is on the development and diffusion of knowledge concerning the local conditions necessary for receiving and propagating the specific infections of disease that nearly all the important sanitary measures of modern times have been based. And it is on a further development of knowledge in the same direction, gained by more systematic, continuous, and coincident investigation, that we shall most successfully protect our race from the pestilences that have hitherto "walked in darkness and wasted at noonday."

It was the extensive and ever extending field of medical science, the complexity of the problems pressing for solution, and still more the individual responsibility of applying the resources at command to the direct treatment of disease, that early disposed medical men to seek each other's counsel, to form groups or clubs for comparison of views and mutual improvement. The manifest advantages of these soon prompted more extended social gatherings, until at the present time a large proportion of the more active members of the profession in every civilized country are participating in municipal, district, National, and International medical organizations.

The aggregate benefit derived from all this active intercourse is beyond easy expression in words. In the more frequent and familiar comparison of cases and views on all professional subjects in the local societies, closer habits of observation and a wider range of thought are induced, while narrow prejudices and bigotry give place to generous rivalry and personal friendship. In the larger gatherings, the formal preparation of papers and reports on a great variety of subjects impels their authors to a wider range of study and greater mental discipline, while the collision with other minds in discussion brings all aspects of the subject to view, enlarging the scope of mental vision, starting new trains of thought, and begetting a broader and stronger mental grasp with purer and nobler aims in life.

I think I am justified in saying that no other one influence operative in human society during the present century has done as much to develop and diffuse medical knowledge, to stimulate its practical and successful application, both in sanitary measures for preventing diesase and in the direct alleviation of suffering at the bedside, and unifying and ennobling the profession itself, as has been accomplished by the aggregate medical society organizations of the world. Yet their capacity for conferring other and perhaps still greater benefits, under proper management, will have become manifest in the near future. And that I may accomplish the chief object of this address, I must ask your indulgence while I indicate some of the more important additional benefits in advancing medical science and saving human life through the instrumentality of our medical society organizations, and the methods by which they may be accomplished.

Every experienced and intelligent practitioner of the healing art is familiar with the fact that all acute general diseases are influenced in their prevalence and severity by seasons of the year, topographical and other conditions of the earth, meteorological conditions of the atmosphere, and the social condition and habits of the people themselves. The most familiar endemics vary annually in the same localities, while the great epidemics that have for ages broken over the comparatively limited boundaries of their habitats only at intervals of years, and extended their ravages from country to country and receded again to the source from which they apparently originated, differ widely in the different periods of their prevalence. But in studying the essential causes of any one of these general diseases and the laws and conditions under which such causes operate, he soon finds certain factors, essential for the solution of his problems, wanting.

For instance, if he wishes to identify the date of the first attack of epidemic cholera in a given locality, and the character of bowel affections immediately preceding, the ordinary statistics of mortality will give him only the date of death, which may have been from one to seven days later, or it may have been preceded by one or more cases that recovered. If he is anxious to determine the reason why the disease, on entering one community, develops with such rapidity that in a few days its victims are found in every grade of the population and in almost every street, while in an other it develops slowly, adhering persistently to particular classes or localities, he may find in the ordinary meteorological records the thermometric, barometric, and hygrometric conditions of the atmosphere, with the direction and the velocity of the winds, but he finds nothing regarding those important though variable elements known as ozone and hydrogen peroxide, active oxidizers; or those nitrogenous products called free and albuminoid ammonia. Neither do the sanitary records give the desired information concerning the composition and impregnations of the soil, or of the organic and inorganic emanations that may arise therefrom.

An adequate knowledge of these absent factors relating to the condition of the earth, air, and water over districts large enough to embrace localities subject to invasions of the epidemics and others known to be exempt, through a sufficient length of time to cover several periods of prevalence and periods of absence alike, is essential for enabling us to comprehend the causes that make one district amenable to the prevalence of a disease and another not, as well as the marked differences in the severity and mode of progress of the same disease at different periods in the same localities and same classes of the people. The same additional knowledge would also furnish the basis for further sanitary measures of the greatest practical value.

And yet it must be obvious that the co-operation of numbers of medical men directly engaged in the field of general practice, with others possessed of more practical facilities for chemical and microscopical research, is necessary for successfully prosecuting such coincident and continuous investigations as would be likely to secure the desired results. Only well-trained general practitioners in every locality chosen for observation could observe and record the date of the initial symptoms of acute general diseases coming under their notice, and at stated intervals collate and report them to a central committee. The daily observations concerning the presence and relative proportion of active oxidizers and of nitrogenous organic elements in the atmosphere and the water would require the selection of one or two experts in chemical and microscopical research for each locality; all making their observations coincidently in time and by uniform methods.

There are included in the organized medical associations of each country the men and materials necessary for prosecuting every well defined line of inquiry; and these associations, by their stated meetings and their facilities for inter-communication and concert of action, present the entire machinery needed and are only waiting for well planned and systematic use.

The tendency to make the permanent medical organizations available for prosecuting work in the directions I have indicated has already been manifested to a limited extent, as may be seen in the formation of the Collective Investigation Committee of the British Medical Association and of the International Collective Investigation Committee, organized during the sitting of the Eighth International Congress at Copenhagen.

An earlier movement more fully of the character I have been endeavoring to explain was made by the American Medical Association in 1875[1] when a standing committee was appointed to establish in a sufficient number of localities regular coincident daily observations and records concerning all appreciable meteorological conditions including organic and inorganic elements found in the atmosphere, and the date of beginning of acute general diseases, and report the results at each annual meeting of the association.

The committee made reports embodying facts of interest and permanent value in 1877[2], in 1879[3], in 1881[4], in 1882[5], and in 1883[6]. The latter report contains among other items a complete tabulated statement of the free albuminoid ammonia in the atmosphere for every day in the year ending Aug. 31, 1883, as determined for the committee by Prof. J. H. Long in connection with the laboratory of the Chicago Medical College. The committee is still prosecuting its work with material in hand for a still more important report at an early day. The greatest difficulty encountered has been to enlist a sufficient number of active practioners in each locality to faithfully record the desired

1 See Trans. American Medical Association, Vol. 26, p. 125.
2 See Trans. American Medical Association, Vol. 28, p. 153.
3 See Trans. American Medical Association, Vol. 30, pp. 38-147.
4 See Trans. American Medical Association, Vol. 32, p. 481.
5 See Trans. American Medical Association, Vol. 33, p. 43.
6 See Journal of American Medical Association, Vol. 2, pp. 85 and 169.

clinical facts and report the results to the committee. But this and all other obstacles can be overcome by persevering and well-directed work.

I trust no apology is needed for having embraced this occasion to attract your attention to the very important question how to make all our Medical Associations more useful in promoting the science of medicine by more complete methods of investigation, especially in directions where the coincident action of several persons in different places, is essential for success.

I fully appreciate the great benefit resulting from the simple mingling of large numbers of medical men in social contact where each is made to hear constantly, whether on the street, in the hotel, or the assembly room, new suggestions, new modes of expression and to observe the physical and mental effects of the various habits and customs of the different peoples, until each one leaves the general gathering with largely increased mental activity and resources as was so happily expressed by Sir James Paget in his address to the Congress of 1881 in London. And I appreciate in a still higher degree the benefits derived from the preparation and reading of papers by individuals and the discussion of important questions in all our assemblies.

But for reasons I have already briefly stated, I hope to see added in every permanent general medical society, two standing committees; one, to whom should be referred for critical examination every communication claiming to embody a new discovery in either the Science or Art of Medicine; and the other should be charged with the work of devising such lines of investigation for developing additional knowledge as require the co-operation of different individuals, and perhaps societies, and of superintending their efficient execution until crowned with success.

If ten or twenty per cent. of the money paid for initiation and membership dues by the members of each society were appropriated and judiciously expended in the prosecution of such systematic and continuous investigations from year to year, it would accomplish more in advancing medical science directly, and indirectly in benefitting the human race, than ten times that amount would accomplish if expended in any other direction.

For it must be remembered that when money is expended for material objects, even for food, clothing, or medicine, such materials feed, clothe, or relieve but one set of needy individuals and are themselves consumed; but the expenditure of money and time in such a way as to develop a new fact capable of practical application either in preventing, alleviating, or curing disease, that *fact* does not like the food or medicine perish with the using, but it becomes literally imperishable. Neither are its benefits limited to one set of individuals, but it is transmitted with the speed of the lightning over the land and under the sea to every civilized people; and whatever benefits it is capable of conferring are as capable of being applied to a million as to one, and of being repeated with increasing efficiency from generation to generation.

It has been tersely and correctly stated that associated action constitutes the characteristic and predominating power of the age in which we live.

It is by associated action that education in its broadest sense, religion, and civilization have been more rapidly diffused among the masses of mankind during the present century, than during any other period of the world's history.

It is by the association of capital, wielded by the associated intellects of the nineteenth century, that highways of commerce have been opened over the valleys, through the mountains, across the deserts, and on the oceans, over some of which the material productions of the nations are borne by the resistless power of steam, and along others the products of mental action are moved with the speed of electric currents, until both time and space are so far nullified that the most distant nations have become neighbors, and the inhabitants hold daily converse with each other from opposite sides of the globe.

Indeed, it is only by means of such of these highways as have been constructed within the memory of him who addresses you, that you have been gathered in this hall from the four quarters of the earth, and through which an account of your doings may be daily transmitted to your most distant homes.

I congratulate you on the fact that the profession you represent has taken the lead of all other professions or classes of men, in rendering available these grand material achievements of the age, for cultivating fraternal relations, developing and interchanging knowledge, and planning concerted action for rendering human life everywhere healthier, happier, and of longer duration.

This is the ninth grand International Congress in regular series within little more than two decades, and let us hope that all its work will not only be done in harmony and good order, but with such results as will add much to the aggregate of human happiness through all the coming generations.

Without tresspassing further on your patience, I must ask your forbearance with my own imperfect qualifications, and your generous assistance in the discharge of the responsible duties you have devolved upon me.

